# Important soil microbiota's effects on plants and soils: a comprehensive 30-year systematic literature review

**DOI:** 10.3389/fmicb.2024.1347745

**Published:** 2024-03-25

**Authors:** Xueling Wang, Yongkuan Chi, Shuzhen Song

**Affiliations:** School of Karst Science, State Engineering Technology Institute for Karst Desertification Control, Guizhou Normal University, Guiyang, China

**Keywords:** significant microbiota, effect mechanisms, ecosystem, functions, stability

## Abstract

Clarifying the relationship between soil microorganisms and the plant-soil system is crucial for encouraging the sustainable development of ecosystems, as soil microorganisms serve a variety of functional roles in the plant-soil system. In this work, the influence mechanisms of significant soil microbial groups on the plant-soil system and their applications in environmental remediation over the previous 30 years were reviewed using a systematic literature review (SLR) methodology. The findings demonstrated that: (1) There has been a general upward trend in the number of publications on significant microorganisms, including bacteria, fungi, and archaea. (2) Bacteria and fungi influence soil development and plant growth through organic matter decomposition, nitrogen, phosphorus, and potassium element dissolution, symbiotic relationships, plant growth hormone production, pathogen inhibition, and plant resistance induction. Archaea aid in the growth of plants by breaking down low-molecular-weight organic matter, participating in element cycles, producing plant growth hormones, and suppressing infections. (3) Microorganism principles are utilized in soil remediation, biofertilizer production, denitrification, and phosphorus removal, effectively reducing environmental pollution, preventing soil pathogen invasion, protecting vegetation health, and promoting plant growth. The three important microbial groups collectively regulate the plant-soil ecosystem and help maintain its relative stability. This work systematically summarizes the principles of important microbial groups influence plant-soil systems, providing a theoretical reference for how to control soil microbes in order to restore damaged ecosystems and enhance ecosystem resilience in the future.

## 1 Introduction

The principle of microbial remediation of the environment is a topic that people have always been working on researching (White et al., 1998; Cruz et al., 2017; Sharma et al., 2022; Verma et al., 2023; Torres-Farradá et al., 2024). Utilizing microbial remediation techniques can not only clean and efficiently reduce environmental pollution but also restore and rebuild ecosystems, maintaining ecosystem stability (Singh et al., 2019; Lu et al., 2023; Saeed et al., 2023). As high-throughput sequencing technology and molecular information technology increasingly develop (Qiang-long et al., 2014; Gray et al., 2015), a lot of new microbial strain species have been found and chosen. By studying their structure and function, we can predict how these microbial strain work to restore ecosystems and clean environment (Liu et al., 2020a; Fernandez Nuñez et al., 2021; Xiao et al., 2021). After analyzing and summarizing current research findings, it can be found that bacteria, fungi, and archaea are important components of the soil microbial community (Fierer, 2017). However, in studies aimed at environmental remediation, the knowledge on how important microbial groups exert their effects is relatively fragmented, and the research content is not detailed enough. It is worth nothing that elucidating the mechanisms by which important soil microbes exert their effects is of significant reference value for restoring damaged ecosystems. Moreover, as the main targets of microbial activity in the soil, the changes in plants and soil will reflect the level and quality of microbial environmental remediation capabilities (Ma and You, 2016; Zhao et al., 2021). Thus, exploring the structure, physiological activities, and impact mechanisms of important microbial groups at the phylum and genus levels can clarify the complex relationships among microbes, plants, and soil. Through using the right and appropriate mechanisms, people can improve the resilience of soil ecosystems against disturbances, increase their functionality, reduce the harm that pollutants cause, and better maintain the stability of ecosystems.

**Figure 1:**
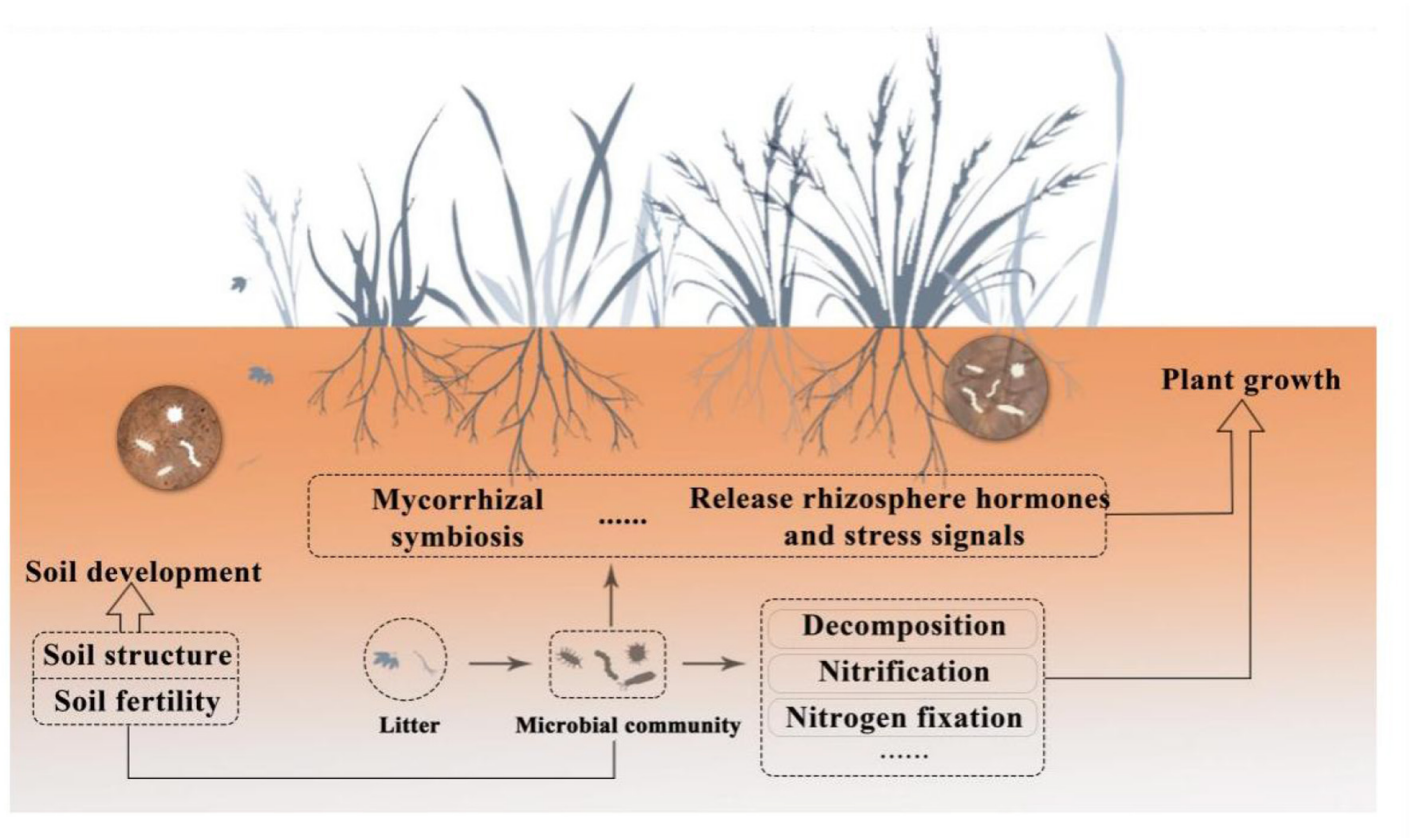
Schematic diagram of the relationship between soil microorganisms, plants, and soil.

According to Nemergut et al. (2013), soil microbial communities are among the most abundant and diverse biological groups in nature. A rich and varied microbial population, comprising bacteria (including actinomycetes), fungi, viruses, archaea, algae, and protozoa, can be found in one gram of soil (Pepper and Gentry, 2015; Islam et al., 2020; Sokol et al., 2022). Among them, bacteria, fungi, and archaea are important components of soil microorganisms (Bayranvand et al., 2021). With 70–90% of the total biomass in the soil, bacteria are the most prevalent microorganisms. Fungi are second only to bacteria in abundance. Both are engaged in nearly all ecological processes that impact soil and plants. Archaea—which inhabit extreme environments—make up a substantial fraction of soil microorganisms. Due to their distinct structural features, they are referred to as the third form of life (Allers and Mevarech, 2005; Ibáñez de Aldecoa et al., 2017). It is helpful to promote further research on microbial diversity based on studying the way archaea affect plants and soil. As opposed to bacteria, fungus, and archaea, algae and protozoa are much less common and have less of an effect on soil and plants (Devi and Soni, 2019). In the plant-soil underground ecosystem, soil microorganisms mainly establish connections with plants in the following three ways (Van Der Heijden et al., 2008): (1) plant residues, including roots, leaves, and other secretions, are the primary source of soil carbon to supply microbes in the soil (Zhang et al., 2022a); (2) soil microorganisms release nutrients through metabolic activities, supporting soil development, promoting plant growth, and maintaining the stability of soil carbon cycling; (3) through mycorrhizal symbiosis (Hayat et al., 2010), releasing hormones, and stress signals (López-Bucio et al., 2007), microorganisms can directly affect plant growth and development. On the other hand, microorganisms can establish soil structure and form soil aggregates through gas exchange and the production of organic acids (Ahmed et al., 2021). Microorganisms can also increase soil fertility by breaking down organic matter and minerals, releasing nutrients, and releasing inorganic compounds (Wang et al., 2021a), as seen in Figure 1. Thus, microorganisms are involved in influencing the ecological processes that affect plants and soil, maintaining the stability of the plant-soil ecosystem (Huet et al., 2023).

Diverse soil microbial groups affect plants and soil through different mechanisms. Common soil bacteria and fungi play important roles in decomposing organic matter, suppressing the growth of pathogens, promoting nutrient cycling, and improving soil texture and structure. For example, a type of bacteria called *Bacillus subtilis* is in the Firmicutes phylum. It can make bacillomycin, organic acids, and antibacterial proteins that stop pathogens from spreading and growing (Zhang et al., 2022b). Another example is the *Rhizobium* genus, which belongs to the Proteobacteria phylum and changes nitrogen gas into ammonia or nitrite that plants can use. It does this by fixing nitrogen, which helps plants grow and encourages nitrogen cycling (Sharma et al., 1993; del Carmen Orozco-Mosqueda et al., 2013; Saidi et al., 2021). Moreover, *Pseudomonas*, a genus within the Proteobacteria phylum, secretes organic acids, which contribute to improving soil structure and maintaining soil moisture (Halverson et al., 2000; Archana et al., 2013). For soil fungi, *Trichoderma* is a genus of fungi in the Deuteromycotina subphylum that can stop pathogenic fungi and bacteria from growing on plants (Marques et al., 2018). According to Chen et al. (2023), arbuscular mycorrhizal fungi (AMF) in the Glomeromycota phylum live in a mutualistic symbiosis with plant roots. This helps plants get more nutrients and grow and develop. Meanwhile, AMF's mycelial network can also encourage soil particle aggregation, which helps to produce soil structure and enhances aeration and water retention in the soil (Beare et al., 1997; Zhang et al., 2022c). On the other hand, in order to preserve the health of the soil and the ecological balance, archaea are crucial to the decomposition of organic matter, the cycling of nitrogen, and the consumption of methane. For instance, methanogenic archaea (MA) and ammonia-oxidizing archaea (AOA) mediate the cycling of carbon, nitrogen, and other elements in the soil to maintain plant growth and health, and they belong to the Euryarchaeota and Thaumarchaeota phylums, respectively (Jung et al., 2020; Ni et al., 2023). Compared to bacteria, fungi, and archaea, algae and protozoa have comparatively less of an effect on plants and soil because they have certain needs for their living conditions. Algae use photosynthesis to build up organic materials in the soil while they reside in damp surface soil. Research has demonstrated that cyanobacteria can fix nitrogen, which qualifies them as biofertilizers (Ammar et al., 2022). Because they consume organic leftovers and decompose in soil that is rich in organic matter, protozoa have the ability to alter the makeup and abundance of other soil microbial communities (Guo et al., 2022). These microbes have a unique living habitat, which makes their abundance variable and highly susceptible to outside influences. Therefore, in this study, algae and protozoa are not regarded as significant microbial groups. Understanding how the above important soil microbial groups affect plants and soil can help with managing them, which will promote plant growth, improve soil quality, and keep the ecosystem stable in the long run.

Numerous studies have been done on certain microorganisms that are found in the core root biome right now, like plant growth-promoting rhizobacteria (PGPR) and plant growth-promoting fungi (PGPF; Raho et al., 2022; Jernigan et al., 2023; Ramakrishnan et al., 2023). In addition, there is also a lot of research on the contributions of MA and AOA to the processes of carbon and nitrogen cycling (Ke et al., 2014; Day et al., 2022). Despite some encouraging findings, it is still unclear how significant microbial communities affect plants and soil and how these processes are systematically understood. To fully understand the intricate interactions between soil, microbial groups, and plants, as well as the potential role that soil microbes may play in enhancing ecosystem stability and function, more research is required. Furthermore, there is still a lack of knowledge about the application of restoring ecosystems to important microbial groups' action mechanisms. Therefore, from the perspective of soil microorganisms, we use the systematic literature review (SLR) method to address the above issues. At the phylum and genus levels of important microbial groups, their mechanisms that affect plants and soil will be summarized, and the latest research progress will be reviewed. We will conduct a systematic review of global research progress on the impacts of important microbial groups on plants and soil from 1992 to 2022 by searching relevant literature in the Scopus database and performing quantitative analysis. By comprehensively analyzing advancements in this field, we aim to provide theoretical references for future research on utilizing microbial principles to restore damaged ecosystems.

## 2 Materials and methods

**Figure 2:**
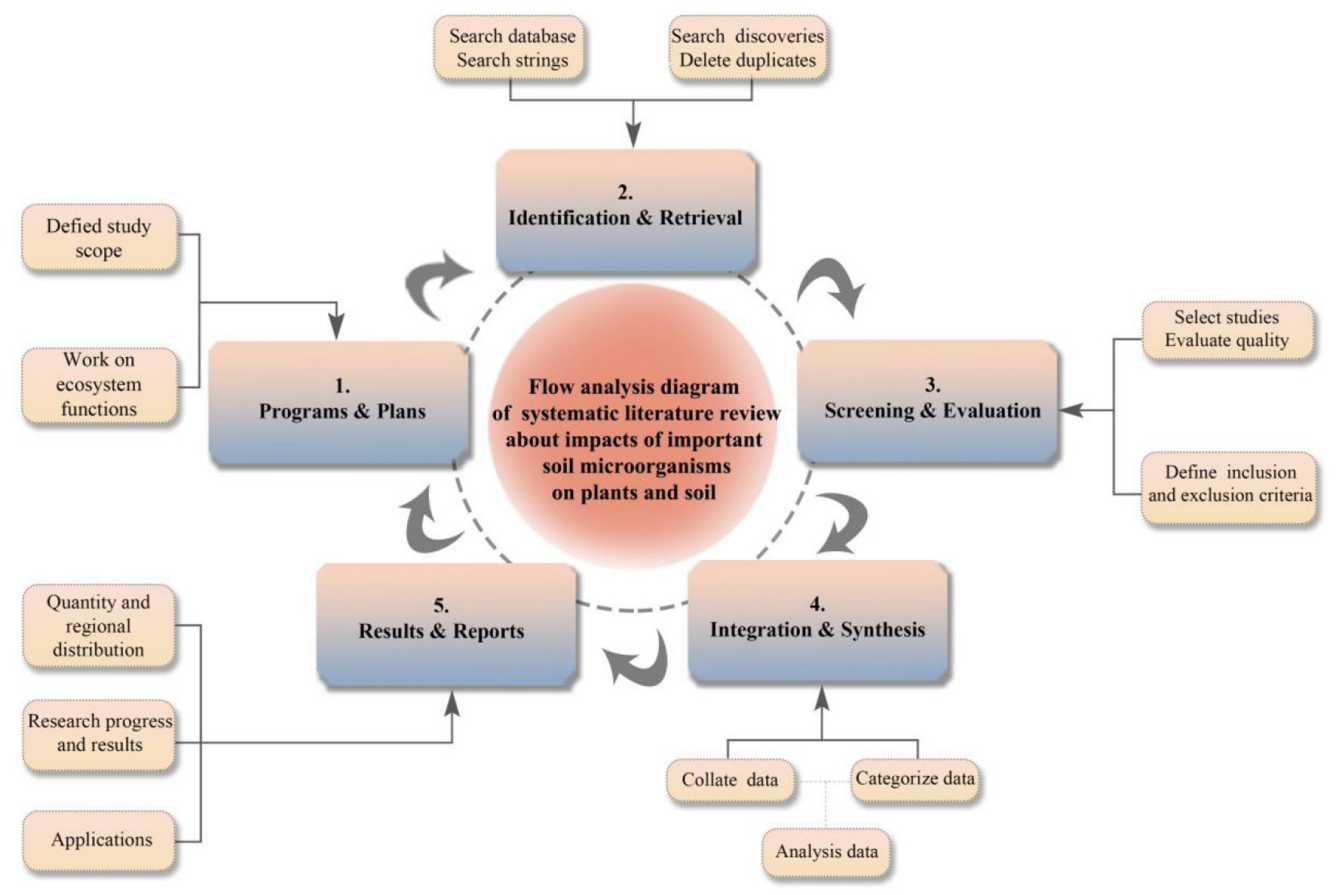
SLR flow framework. Begin with Step 1 and end with Step 5.

SLR is a method based on existing literature to study specific fields. It has the advantages of high relevance to the topic and clear predictions for future vision (Tikito and Souissi, 2019). At the same time, it is an interdisciplinary approach that can be used to summarize the research status of specific topics (Burgers et al., 2019). When searching for literature, entering the correct keywords in the search bar and determining the important steps of the search can minimize the loss of literature information. In this study, we adopted the method of systematic review to organize published literature and scientifically evaluate the impact of soil microorganisms on plants and soil, as shown in the flow framework in Figure 2.

### 2.1 Programs and plans

This study is based on articles from the Scopus database. Scopus is currently the largest abstract and citation database in the world, with the widest coverage, and it maximizes the search for published literature. The literature was screened based on the flow framework, and the research scope was determined based on the selected thematic literature to address research questions and summarize existing progress and research findings.

### 2.2 Identification and retrieval

The second step in the flow framework is the retrieval stage, where relevant articles on the impact of soil microorganisms on plants and soil worldwide are searched in the Scopus database. Keywords such as “soil microorganisms,” “plants,” and “soil” are entered in the search box, and Boolean logic operations are performed. After the initial search, a total of 28,972 records were obtained. Subsequently, review studies unrelated to research progress were excluded, and duplicate articles were removed, resulting in 17,217 articles. Next, abstracts were manually screened using keywords such as “effects, impact, actions, and mechanisms” to narrow down the selection, resulting in 1,959 articles as the preliminary screening results, as shown in Table 1.

**Table 1:** Searching the database for terms about the topic literature.

**Databases**	**Boolean logic operation topic search strings**	**The number of articles initially retrieved**	**The number of articles after initial screening**	**Topic words related to research branches**	**The number of articles in the in topic scope**	**Retrieval date**
Scopus	“soil microorganism” AND “plants” AND “soil”	28,972	1,959	“bacterium” or “bacteria”	161	November, 2023
				“fungus” or “fungi”	64	
				“archaea”	15	
					Total: 240	

### 2.3 Screening and evaluation

One thousand nine hundred and fifty-nine articles were evaluated, and we manually selected literature relevant to our research topic in order to further narrow down the scope of the study. Inclusion and exclusion criteria were used as the basis for assessing the validity of the articles. The inclusion criteria are as follows: (1) The topic words “bacterium or bacteria,” “fungus or fungi,” “archaea,” or similar terms exist or at least appear in the titles, abstracts, or keywords of the literature. (2) The main subject of the research must be soil bacteria, fungi, or archaea. (3) The objects of the research must be plants and/or soil. (4) The study must be related to the mechanisms of the impact of important microbial groups such as bacteria, fungi, and archaea on plants and soil. The exclusion criteria are: (1) The topic words “bacterium,” “fungus or fungi,” “archaea,” or similar terms do not appear in the titles, abstracts, or keywords of the literature. (2) The main subject of the research isn't soil bacteria, fungi, or archaea. (3) The objects of the research aren't plants and/or soil. (4) The study does not primarily focus on the impact mechanisms of important soil microbial groups such as bacteria, fungi, and archaea on plants and soil.

Two hundred and forty articles were obtained according to the inclusion and exclusion criteria in total. Table 1 illustrates the publications and amounts related to the effects of bacteria, fungus, and archaea on plants and soil (*n* = 161, *n* = 64, and *n* = 15, respectively). To gather secondary literature for citation references as publications within the scope of this study, a snowballing strategy was also employed, which involves reading pertinent papers in depth (Greenhalgh and Peacock, 2005). By employing this technique, 26 more articles were found, bringing the total number of articles to 266. After that, the different kinds and amounts of literature were tallied: 234 journal articles, 24 conference papers, seven book chapters, and one short survey.

### 2.4 Integration and synthesis

In this step, a thorough analysis of the thematic content and research methods of the 266 articles was conducted. The content of the literature was then organized and classified, and the relevant research progress was analyzed and summarized. This stage reviewed existing achievements to help us clarify the specific mechanisms by which important microbial communities affect plants and soil, thus laying a theoretical foundation for their better application in promoting ecosystem functionality.

### 2.5 Results and reports

This stage is the last step of the entire process. After completing the aforementioned steps 1–4, we will present the final results and conclusions in this stage. An analysis of the 266 articles obtained during the retrieval phase will be conducted to determine the annual distribution of publications, global contributions by country, and research trends for the thematic keywords. The research findings will be detailed in Sections 3.1–3.3 of this study. Sections 3.4 and 3.5 will provide a summary of the mechanisms by which important microbial communities affect plants and soil, as well as their current application in ecosystem functionality.

## 3 Research results

### 3.1 Publications and annual distribution

**Figure 3:**
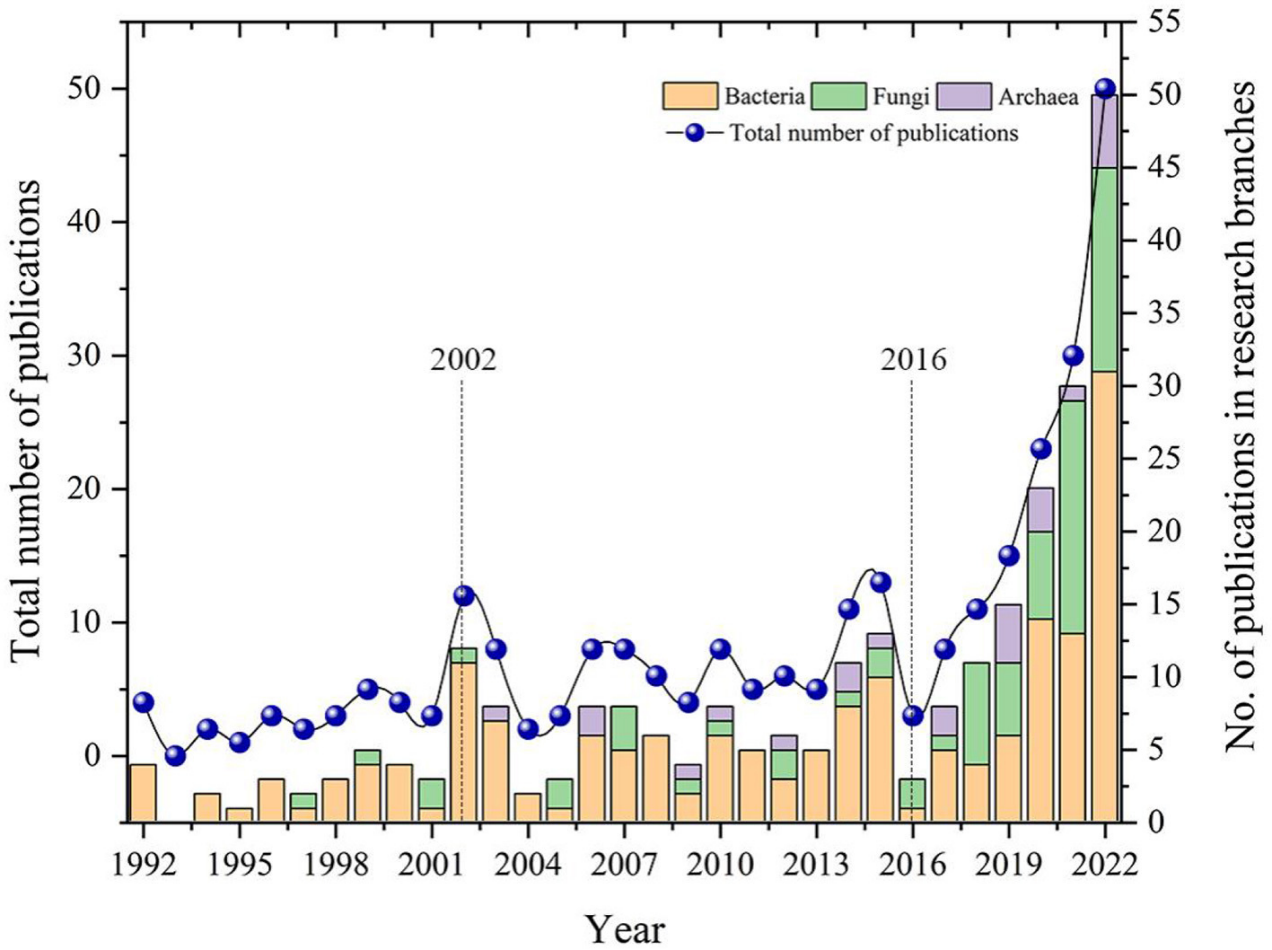
Distribution of global publications on the impacts of bacteria, fungi, and archaea on both plants and soil from 1992 to 2022.

The 266 pieces of literature that were selected were statistically analyzed, and the results are shown in Figure 3. This shows how publications about the effects of large microbial populations on plants and soil changed around the world from 1992 to 2022. Based on the graph, it can be observed that the number of articles on this topic is generally increasing and is likely to continue to grow. Since the 1990's, global research on soil microbiology has undergone rapid changes, driven by technological advancements and increased attention to soil microorganisms. The protection and sustainable use of soil microbial diversity were formally proposed in 2002 at the 6th Conference of the Parties (COP6) of the International Convention on Biological Diversity (Chandra and Idrisova, 2011; Zhu et al., 2017). This proposal raised awareness of the significance of soil microbiology in environmental science and agriculture. Afterwards, more research is being done on soil microbiology because more new microbial species are being found and molecular techniques for studying soil microbiota are getting better (Keller and Zengler, 2004; van Elsas and Boersma, 2011), and research on soil microbes has entered a growth phase. In 2016, the United States launched the “National Microbiome Initiative,” aimed at exploring the functions of microbiomes in the environment and providing insights and solutions to major issues facing the twenty-first century, such as agriculture, energy, climate, and the environment. In the same year, advancements in high-throughput sequencing technology and bioinformatics tools made it possible to conduct more comprehensive research on the diversity and functionality of soil microbes (Ciancio et al., 2016; Esposito et al., 2016). This has expanded the application field of microbiology and propelled it into a period of rapid development.

In terms of research content branches, there is a significant overall increase in the study of bacteria and fungi related to their impact on plants and soil. In contrast, the number of research papers on the impact of archaea on plants and soil shows slower growth. This phenomenon may be due to our limited understanding of archaea at present, as research in this area is still in the exploratory stage, resulting in a slow growth in the number of relevant research papers (Baker et al., 2020). Additionally, the National Microbiome Initiative implemented in the United States has promoted the development of microbiology research (Alivisatos et al., 2015), and the publication volume related to this topic has shown even faster growth since 2016.

### 3.2 Global distribution of contributors

**Figure 4:**
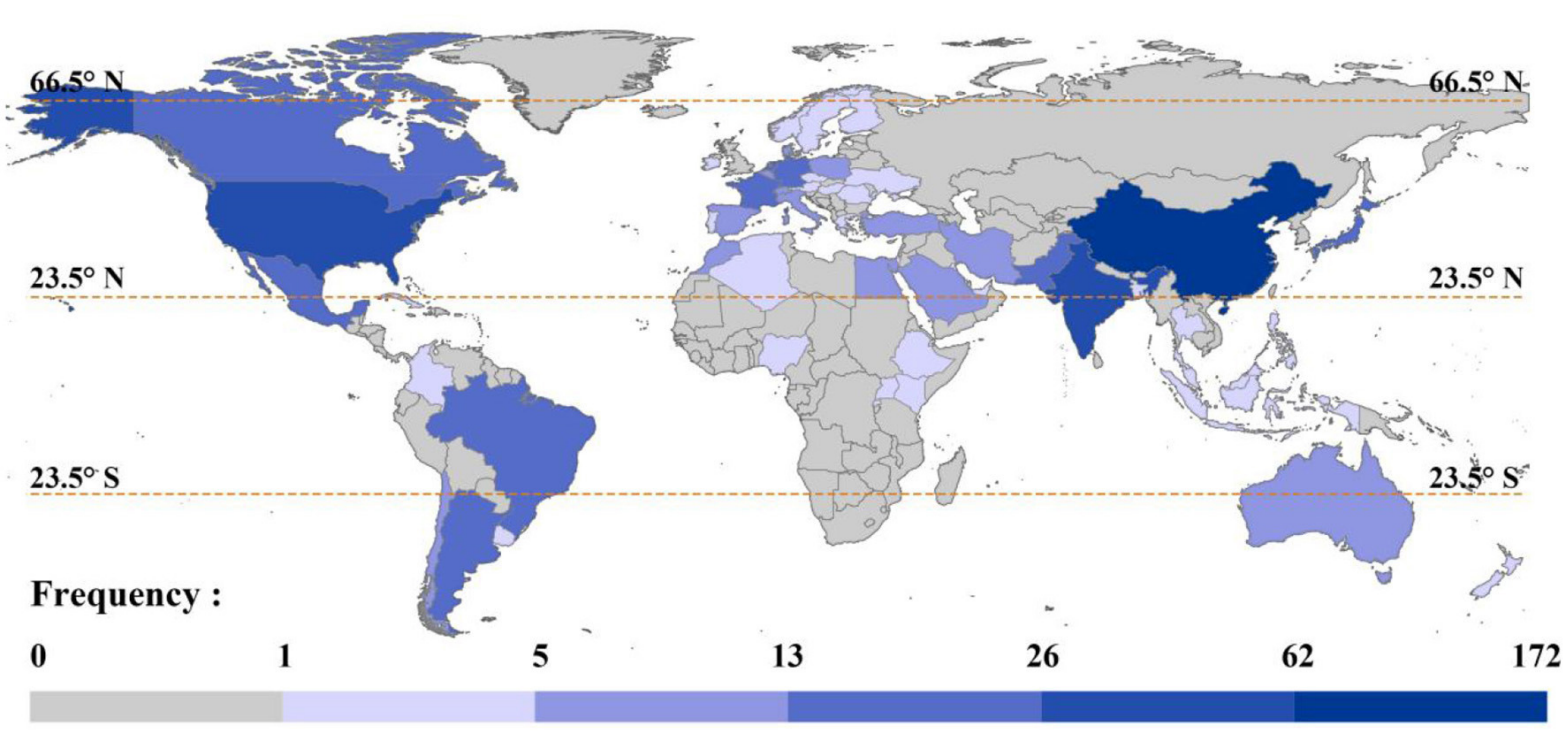
Global distribution of countries contributing to research. The darker the color, the more articles contributed, and vice versa.

An analysis of national affiliations was conducted on 266 articles, and the results are shown in Figure 4. In terms of the global geographical distribution of research on the impacts of important microbial groups on plants and soil, China shows the highest level of research focus, with a frequency of 172 occurrences, making it the most prominent country in this field. The United States ranks second with 62 occurrences. India comes in third with a score of 41, ahead of Germany, France, and the Netherlands, all of which have scores above 20. This result reflects the high importance that China, as an agricultural powerhouse, places on research on soil microorganisms and the significant increase in attention given to soil and ecosystem conservation since 2016 (Wen et al., 2022).

### 3.3 Research focus and trends

**Figure 5:**
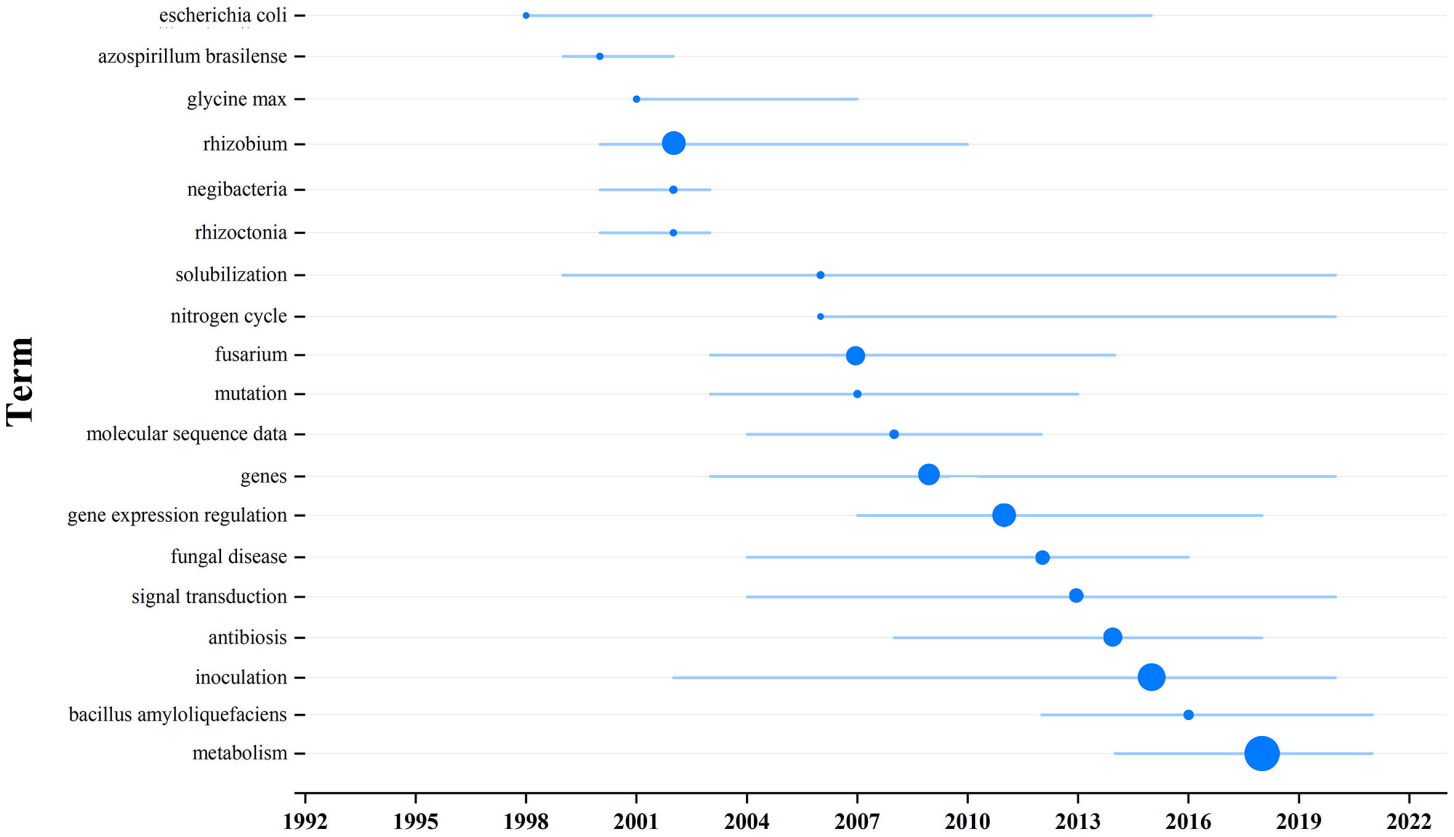
Topic term research trend chart. The larger the node, the higher frequent occurrence the keyword.

Visualize the main terms relevant to the topic from the selected 266 pieces of literature, and topic words and trends can be seen in Figure 5. The keywords and their frequencies are detailed in Table 2. The charts show that from 2002 to 2016, most of the research on how soil microbial communities affect plants and soil was focused on three words: “rhizobium,” “gene expression regulation,” and “inoculation.” All three of these words were used more than 20 times. Following closely were “fusarium,” “genes,” and “antibiosis,” with frequencies exceeding 15. After 2016, research focused on “(microbial) metabolism” with frequencies of 50, which is also the main research content of this article.

**Table 2:** Topic keywords and frequency.

**Year**			**Keyword**	**Frequency**
**Initial**	**Middle**	**Final**		
2000	2002	2010	Rhizobium	22
2003	2007	2014	Fusarium	16
2003	2009	2020	Genes	18
2007	2011	2018	Gene expression regulation	21
2008	2014	2018	Antibiosis	17
2002	2015	2020	Inoculation	33
2014	2018	2021	Metabolism	50

### 3.4 Important soil microbiota's effects on plants and soils

#### 3.4.1 The mechanisms of soil bacterial impact on plants and soil

**Figure 6:**
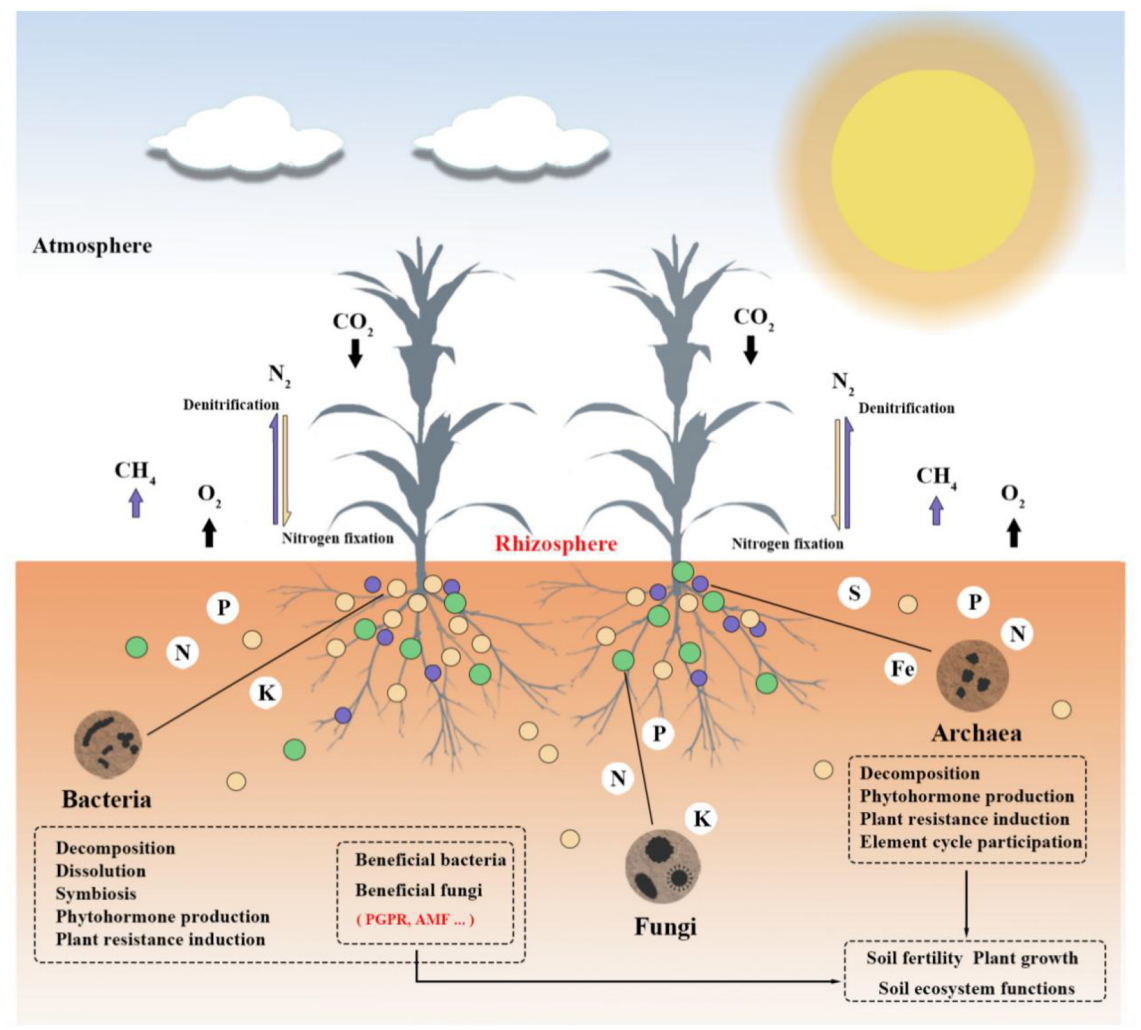
Conceptual map of mechanisms of action of important soil microbial communities.

The most prevalent microbial population in soil, soil bacteria, have a major influence on plant health, soil texture, the nutrient cycle, and biodiversity. As seen in Figure 6, they primarily affect soil structure and plant growth in the following ways: (1) By secreting enzymes that convert organic materials into nutrients that plants may use, bacteria take part in the breakdown of organic matter in the soil. For example, different proteases and polysaccharide hydrolases can be released by *Bacillus subtilis* and *Bacillus licheniformis* (Bais et al., 2004; Lee et al., 2022). *Cellulomonas uda* is a wet cellulose bacterium belonging to the *Cellulomonas* genus that can break down complex macromolecular organic materials into simpler compounds (Pérez-Avalos et al., 2008; Ontañon et al., 2021). Bacteria are also frequently employed to break down organic contaminants in soil. As an instance, phenolic compounds can be broken down by the *Flavobacterium* genus (Larsbrink et al., 2016), and polycyclic aromatic hydrocarbons (PAHs) can be broken down by the *Bacillus* and *Pseudomonas* genera (Saeed et al., 2022). These soil bacteria's decomposition processes contribute to the soil ecosystem's balance, soil environment purification, and soil quality improvement. (2) Soil bacteria aid in the cycling of nutrients, such as potassium, phosphorus, and nitrogen. *Bacillus mucilaginosus Krass*, for instance, has the ability to break down feldspar and convert potassium components that are insoluble into soluble nutrients (Ahmad et al., 2016; Tallapragada and Matthew, 2021). Some types of soil bacteria, including those genera *Erwinia* (Kim et al., 1998; Maldonado et al., 2020), *Pseudomonas* (Alsohim et al., 2014; Oteino et al., 2015; Hamdali et al., 2021), *Agrobacterium* (Belimov et al., 1999; Yu et al., 2011; Zheleznyakov et al., 2022), and *Bacillus* (Ramírez and Kloepper, 2010), can change insoluble phosphate into soluble phosphorus that plants can use, which helps plants grow. (3) Beneficial bacteria can form symbiotic relationships with plants, providing nutrients to promote their growth and development. For example, *Rhizobium* forms symbioses with legume plants, while phosphorus-solubilizing microorganisms form symbioses with plant roots. They change nitrogen and phosphorus that plants can't directly absorb into forms that plants can use for growth through nitrogen fixation (Chabot et al., 1996; Chebotar et al., 2001; Tokala et al., 2002; Tilak et al., 2006; Figueiredo et al., 2008) and phosphorus solubilization actions (Chabot et al., 1993; Purnomo et al., 2005; Badawi, 2010; Turan et al., 2012; Berde et al., 2021; Lelapalli et al., 2021), respectively. (4) Soil bacteria can also suppress pathogens and reduce the occurrence of plant diseases. For example, *Streptomyces* (Bressan and Figueiredo, 2008; Cordovez et al., 2015; Guo et al., 2020) and *Bacillus subtilis* (Li et al., 2013; Sidorova et al., 2018) can produce antibiotics to inhibit pathogens. *Bacillus* can make plants produce antimicrobial peptides, antioxidants, and other chemicals that help plants defend themselves against pathogens and stop their growth (Chakraborty et al., 2006; Ren et al., 2012; Revilla-Guarinos et al., 2014; Ahmed et al., 2022). *Rhizobium* can make secondary metabolites that can affect plant signaling systems in a direct or indirect way. This can cause plants to make good bacteria and become resistant to disease (Zehnder et al., 2001). Plants can protect themselves and get induced systemic resistance from molecules made by *Pseudomonas* bacteria. These molecules include salicylic acid and volatile organic compounds (Bigirimana and Höfte, 2002; Pieterse et al., 2003; Siddiqui and Shaukat, 2003). *Bacillus* (Huang et al., 2014, 2015) and *Pseudomonas* (Karnwal, 2011) bacteria have been widely used in biocontrol and are known as biocontrol bacteria. (5) Some soil bacteria can produce plant hormones such as indole acetic acid (IAA), gibberellins, and cytokinins. They promote plant growth by influencing physiological processes such as plant growth, flowering, and fruit development. *Streptomyces* and *Micromonospora* can produce gibberellins (Bottini et al., 2004), and PGPR-like *Bacillus* can produce IAA (Pishchik et al., 2002; Zou et al., 2010). Research has shown that *Bacillus subtilis WW1211* can promote plant growth by regulating coenzyme biosynthesis and redistribution (Wang et al., 2021b). (6) Soil bacteria can also improve soil structure and texture. Bacteria like *Pseudomonas aeruginosa, Bacillus subtilis*, and *Chromobacterium violaceum* can work together to make polysaccharide adhesives that stick to soil particles, help soil aggregates form, improve soil structure, make soil more aerate and hold on to water, and make soil more fertile (Martens and Frankenberger, 1992). In fact, a single soil bacterium can perform multiple functional roles simultaneously. Some examples are *Pseudomonas*, which can help with nutrient cycling and plant defense mechanisms for biocontrol while also helping plants grow (Jacobson et al., 1994); *Streptomyces*, which can make antibiotics to stop pathogens; and they produce gibberellins, which can help plants grow (Solá et al., 2021). To sum up, soil bacteria play a collective role in promoting plant growth, shaping soil structure, and participating in ecological processes. Their interactions with plants and other microorganisms have significant impacts on plant and soil ecosystems. The interplay of these mechanisms collectively maintains the balance and stability of ecosystems.

External environmental factors frequently drive the mechanisms by which soil bacteria affect plants and soil. People often use environmental factors to induce bacteria to promote certain ecological processes in order to enhance ecosystem functionality. For example, the cadmium-induced rapeseed *Xanthomonas campestris* has shown adaptive resistance and cross-resistance to Zn. This characteristic can alleviate the toxicity of Zn in soil to plants, reduce the harm of metal to plants, and maintain the functionality of soil ecosystems (Banjerdkij et al., 2003). In addition, adding bioorganic fertilizers can stimulate the quantity of *Pseudomonas* in the soil. *Pseudomonas* can play a role in biological control and enhance the suppression ability of plant diseases (Tao et al., 2020). Another example is that biochar made from tobacco straw can alter the rhizosphere bacterial community, allowing growth-promoting bacteria to flourish and promote cherry growth (Yang et al., 2022). However, remarkably, adding organic fertilizers can also stimulate the growth of pathogenic bacteria (Tariq et al., 2022). Thus, when artificially inducing certain ecological processes, it is important to have a thorough understanding of the types of bacteria involved. Deepening the understanding and research of these mechanisms of soil bacteria can help in artificially regulating ecological processes and applying them to agricultural production. This will better harness the role of bacteria in enhancing ecological functionality and promoting beneficial ecological functions and services for humans.

#### 3.4.2 The mechanisms of soil fungi's impact on plants and soil

Although there are some similarities and distinctions, the methods by which soil fungi affect plants and soil are similar to those of bacteria. As seen in Figure 6, they mostly have the following effects on soil and plants: (1) Fungi aid in the breakdown of soil organic matter by reducing complex organic molecules to simpler ones and hastening the organic matter's mineralization process. For example, *Paecilomyces lilacinus 112* can secrete hydrolytic enzymes to break down organic matter (Constantin et al., 2022). (2) Soil fungi can convert nutrients such as phosphorus and iron, which plants cannot directly obtain, into available chemical substances (Cao et al., 2015). For example, the *Penicillium* genus can break down insoluble phosphates (Whitelaw et al., 1999; Wakelin et al., 2006; Gómez-Muñoz et al., 2018; Sang et al., 2022), which boosts crop output. (3) In order to provide plants with nutrients and water, soil fungi establish a mycorrhizal symbiosis with plant roots and mycelial networks are responsible for achieving this symbiotic relationship. Fungi that require organic carbon from plants for energy, which is produced through photosynthesis, such as AMFs, are essential for ecological processes. With 80% of terrestrial plants, AMF can create an arbuscular mycorrhizal symbiosis and help plants grow, take up water and nutrients, and maintain healthy soils by regulating the cycling of nutrients (Lee et al., 2014; Mansur et al., 2019). Through competing for nutrients and space, the mycorrhizal symbiosis between AMF and plant roots can make plants more resistant to disease, fight soil-borne pathogens, make plant communities better able to handle stress from outside sources, and improve plant growth and soil quality. This helps restore ecosystems that can take care of themselves (Barea et al., 2011; Pickett et al., 2022). According to Bianciotto et al. (2018), AMF is a crucial component of ecosystems, and its scarcity or absence can reduce the ecosystems' ability to function as efficiently as possible. Hence, using AMF can recover damaged mining ecosystems, which has been applied extensively (Wang, 2017). (4) Some soil fungi can control diseases by producing antibiotics or metabolites harmful to plant pathogens. For example, the *Trichoderma* genus releases volatile organic compounds that activate the plant's defense mechanism against powdery mildew (Yao et al., 2016; Lazazzara et al., 2021; Cabral-Miramontes et al., 2022). They protect plant health by competing for nutrients with pathogens or inhibiting their growth. In addition, some soil fungi mediate the production of signaling molecules to activate plant defense responses, induce the plant's immune system, enhance its resistance to pathogens, and reduce the occurrence of diseases. For instance, endophytic insect-pathogenic fungi can affect wheat growth through immune responses and related hormone signaling pathways (González-Guzmán et al., 2022). *Trichoderma atroviride* is a fungus that lives inside strawberry trees and protects them from plant pathogens by releasing acids that break down cellulase, proteins, and other compounds that kill pathogens (Martins et al., 2022). (5) Most soil rhizosphere fungi can secrete auxins, such as IAA, to promote plant growth. In the Takalar District, 17 different types of fungi were found to produce IAA. These included *Trichoderma, Fusarium, Aspergillus, Penicillium*, and *Gliocladium* (Larekeng et al., 2019). Among them, *Trichoderma* produced the most IAA. In summary, soil fungi play a significant role in providing nutrients to plants, enhancing plant immune systems, decomposing organic matter, and improving soil structure. Through their interactions with plants and other soil microorganisms, they have important effects on plant and soil ecosystems. The synergistic effects of these mechanisms maintain the balance and stability of soil ecosystems.

However, the impact of soil fungi on plants and soil is not always beneficial. Research has shown that parts of fungi are a major source of pathogenic organisms. For example, *Fusarium oxysporum*, a devastating soil-borne pathogen, is a major source of plant diseases (Rekah et al., 2001; Lisboa et al., 2015; Goncharov et al., 2022). *Phytophthora capsici*, a polyphagous plant pathogen, can infect dozens of plant species (Saltos et al., 2022). *Plectosphaerella melonis*, a common pathogen of cucurbits in the United States and Spain, can infect plants such as gourds, peppers, tomatoes, basil, and parsley (Tsekhmister and Kyslynska, 2022). As a result, it is essential to differentiate between beneficial fungi and pathogenic fungi when studying the mechanisms of fungal impact on plants and soil.

#### 3.4.3 The mechanisms of soil archaeal impact on plants and soil

**Figure 7:**
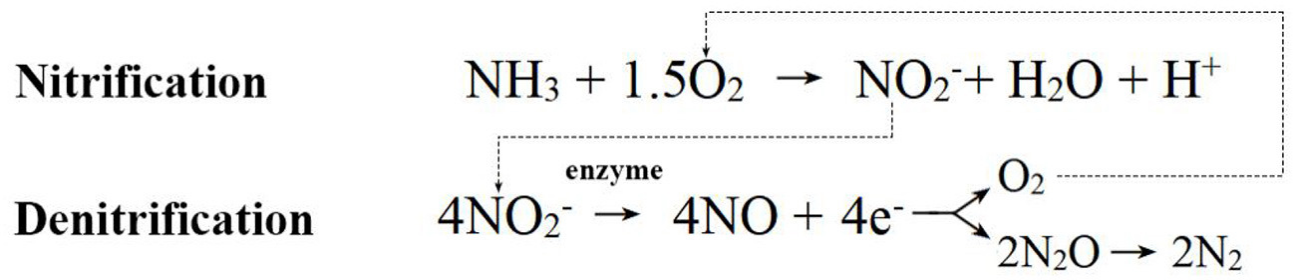
Diagram of ammonia-oxidizing archaea mechanism.

Compared to bacteria and fungi, our understanding of archaea is relatively limited, and current research still mainly focuses on isolating and identifying new archaeal taxa. However, existing research has shown that the roots and rhizosphere of plants create both anaerobic and aerobic microhabitats. These areas are perfect for MA and AOA organisms because they help release methane and are involved in the nitrogen cycle (Bomberg and Timonen, 2009; Pump and Conrad, 2014; Moissl-Eichinger et al., 2018). Consequently, as seen in Figure 6, soil archaea also affect plants and soil in different ways: (1) The decomposing activity of archaea can convert organic matter into nutrient substances that plants can absorb and use, promoting soil nutrient cycling. As an example, AOA can break down organic micropollutants in riverbanks, turning them into small molecules that help the cycle of nutrients and affect plant growth (Zhao et al., 2022). (2) Archaea can facilitate the cycling of substances such as nitrogen, phosphorus, sulfur, and carbon, as well as produce Fe carriers, providing the required nutrients for plant growth. For example, AOA changes ammonia into nitrite during aerobic conditions. During anaerobic conditions, they work with nitrite-oxidizing bacteria to reduce nitrite to nitrogen dioxide during denitrification. This changes how nitrogen is changed on Earth, as seen in Figure 7 for the process (Li et al., 2020; Kraft et al., 2022; Martens-Habbena and Qin, 2022). Archaea provide plants with the necessary nitrogen source through nitrification, promoting plant growth and development. Meanwhile, they also reduce nitrogen gas through denitrification, reducing nitrogen elements in ecosystems. These two processes interact with each other, regulating the nitrogen cycling process in ecosystems and maintaining ecosystem stability. For instance, the new DPANN archaea found in hydrothermal vents take in nitrogen and sulfur and break down amino acids and nucleic acids in the environment. This helps the cycling of nitrogen, phosphorus, and other elements (Cai et al., 2021). MA, on the other hand, turns compounds into methane in the absence of oxygen, which helps the cycling of carbon in nature (Evans et al., 2018). Moreover, halophilic archaea exhibit functional characteristics related to phosphorus and iron production, and they may promote plant growth by enhancing the ability of plants to uptake iron (Dave et al., 2006; Al-Mailem et al., 2010). (3) Archaea can produce plant hormones such as IAA, which affect plant growth. For instance, *Sulfolobus acidocaldarius*, a type of thermophilic microorganism, has been seen to make 1,000 times more IAA than other plant extracts (White, 1987). Additionally, metagenomic analysis of marsh vegetation provides evidence that archaea carry genes for synthesizing auxins, further supporting the notion that archaea can promote plant growth. (4) Archaea activate plant defense mechanisms in multiple ways, enhancing immune responses and protecting vegetation health. For example, archaeal volatile organic compounds (VOCs) can induce resistance in plants. According to research by Ryu et al. (2003) and Song et al. (2019), *Nitrosocosmicus oleophilus MY3* produces VOCs that aid in the growth of *Arabidopsis thaliana*. This suggests that the mechanisms by which archaea promote plant growth may be similar to those of PGPR. Also, archaea can talk to bacteria through intercellular signaling, and they do this by making chemicals like N-acyl-L-homoserine lactones, which affect plant growth and make plants defend themselves (Gao et al., 2019). Besides, archaea can help plants adapt to abiotic stress environments. Some *Euryarchaeota* genus members, like *Methanosarcina*, can stabilize the harmful metal mercury (Hg), which means that plants can still grow even when heavy metals are present (Ma et al., 2019; Zhang and Wang, 2020); Halophilic archaea can alleviate salinity, enhance crop tolerance, promote plant growth, and reduce the impact of environmental stress (Naitam et al., 2023). In general, soil archaea have big effects on plant growth and soil quality. They do this by breaking down organic matter, nitrifying and denitrifying, making plant hormones, and starting up plant defense systems. They play crucial roles in soil ecosystems, maintaining soil health and plant-soil ecosystem stability.

### 3.5 Application of the mechanisms by important microbial groups in restoring ecosystems

#### 3.5.1 Remediation of contaminated soils

Microorganisms can be used to purify soil contaminated with heavy metals and organic compounds through their processes of dissolution and degradation, which improves soil quality and promotes ecosystem functionality to restore damaged ecosystems and a clean soil environment. For instance, functional bacterial strains can remediate contaminated soil. Researchers have found that the bacteria *Paracoccus aminovorans HPD-2* and *Azotobacter chroococcum HN* can break down PAHs into smaller inorganic compounds, which lowers the amount of organic pollution in the soil (Wang et al., 2023). Haloarchaea colonized in the rhizosphere of halophytes, in cooperation with other rhizobacteria, can effectively degrade crude oil. Utilizing these rhizosphere microbial mechanisms can remediate oil-contaminated, high-salt environments. Additionally, some good bacteria and fungi can help plants fight off pathogens, lessen the damage that metals do to plants, increase the production of hormones and enzymes that help plants grow, and work with plants to clean up polluted soils (Ma et al., 2016). As an example, gene expression is changed by plant growth-promoting bacteria (PGPB) such as *Bacillus paranthracis NT1* and *Bacillus megaterium NCT-2* to help *Solanum nigrum L*. get rid of cadmium (Cd), making Cd less harmful to plants and opening the door for PGPB and plants to work together to clean up heavy metal-contaminated soils (Chi et al., 2022). AMF that lives in harmony with plants can stop uranium from moving through soils and plant roots, which stops uranium from moving from roots to shoots and effectively cleans up soils that are contaminated with this metal (Fomina et al., 2020; Wang et al., 2022a). Some types of *Brevibacillus* can make IAA, which, along with AMF, pulls Zn out of the growth medium. This makes Zn-contaminated soils less toxic and helps plants grow (Vivas et al., 2006a,b). Through various mechanisms, these microorganisms efficiently remediate contaminated soils in an environmentally friendly manner, greatly benefiting ecosystem functionality.

#### 3.5.2 Biological fertilizers and microbial agents

In agricultural production, based on the principle of microorganisms promoting plant growth, beneficial microorganisms can be cultured *in vitro* and made into biological fertilizers and microbial agents, which can be applied to improve crop yield, promote local ecosystem functions when needed, and assist in the restoration and rebuilding of ecosystems. For instance, some beneficial microorganisms can be used to produce biological fertilizers: nitrogen-fixing bacteria can convert atmospheric N_2_ into NH_3_ that can be utilized by plants, reducing the use of nitrogen fertilizers in agriculture (Shantharam and Mattoo, 1997; Urquiaga et al., 2012; Smercina et al., 2019; Hu et al., 2021); *Bacillus subtilis N11 with* strong plant colonization ability can effectively control banana wilt disease and serve as a new type of bio-organic fertilizer (Zhang et al., 2011); *Rhizobium* and *Pseudomonads* genera can be used as natural biological fertilizers to promote plant growth (Liu et al., 2020b); Beneficial microorganisms can also be selected and cultured *in vitro* to produce functional strains that can be expanded for use as microbial agents. Microbes like the members of *Bacillus* genus and nitrogen-fixing bacteria can help woody cuttings stay alive and grow in nurseries. They can also help them avoid pathogens and encourage lateral branching (Plugatar et al., 2019). *Penicillium* can break down phosphates, make gibberellins and plant growth regulators to help plants grow, and be used as a bio-inoculant to boost crop yield (Nunes et al., 2022). Some non-pathogenic fungi can also be made from nanoparticles that can effectively control plant diseases, kill bacteria, and lower the toxic effects of metals in the soil. These nanoparticles are commonly used in agriculture as nano-insecticides, nano-fungicides, and nano-herbicides (Alghuthaymi et al., 2015; Cao et al., 2020). However, as certain organic fertilizers can also promote the growth of pathogenic fungi, caution should be exercised when using biofertilizers (Tariq et al., 2022). This method should only be employed properly and efficiently after a thorough understanding of the characteristics and processes of microorganisms.

#### 3.5.3 Biopesticides and nitrogen-phosphorus removal

Utilizing the characteristics and mechanisms of microorganisms, we can produce pesticides and eliminate nitrogen and phosphorus, which not only maintains the health of vegetation and enhances vegetation productivity but also environmentally and economically purifies the environment, effectively maintaining the stability of the ecosystem. Some strains from the genera *Pseudomonas, Bacillus*, and *Trichoderma* have been good at controlling various fungal diseases to reduce the damage that pathogens cause to plants. For example, (a) *Arabidopsis thaliana* plants that are colonized with *Pseudomonas fluorescens* are protected from pathogen invasion (Wang et al., 2022b); (b) *Bacillus amyloliquefaciens hmb33604* is resistant to potato late blight (Fan et al., 2021); (c) the strain of *Trichoderma* genus *LZ42* releases volatile organic compounds that inhibit tomato seedling wilt (Rao et al., 2022). Additionally, the antagonistic interactions between different microbial species can also suppress the growth of pathogens. For example, the antagonistic effect between *Streptomyces violaceus G10* and *Fusarium oxysporum* effectively controls plant diseases (Getha and Vikineswary, 2002); the combination of *Penicillium* and *Bacillus* genera can antagonize the infection of *Fusarium oxysporum* in banana plants (Win et al., 2021). These beneficial microorganisms can be formulated into biological pesticides for the biocontrol of plant diseases, reducing the likelihood of vegetation being infected by pathogens. In terms of eliminating nitrogen and phosphorus elements, microorganisms can reduce environmental pollution (such as water eutrophication) through degradation and participation in element cycling, which indirectly promotes plant growth and maintains the stability of the ecosystem. For instance, AOA nitrification and denitrification processes reduce wastewater nitrogen levels, which aids in the resolution of water pollution issues (Yang et al., 2021). According to Kurt et al. (2017), this technique has also been applied to clean industrial wastewater and encourage the sustainable use of water resources. However, due to their slow growth and sensitivity to their surroundings, AOA can only survive in environments with high levels of ammonia and nitrogen, which indicates that further investigation and study are required (Hu et al., 2023). Under aerobic circumstances, biological phosphorus removal can be accomplished via the physical adsorption and precipitation of microorganisms (Massoompour et al., 2022). Based on the above content, the principles of microorganisms provide us with an effective way to maintain ecosystem stability and environmental cleanliness.

## 4 Discussion

### 4.1 Comparison and limitations

In previous studies, in-depth reviews have been conducted separately on the relationship between soil microorganisms and plants, as well as soil microorganisms and soil (dos Santos and Maranho, 2018; Etesami et al., 2021; He et al., 2022; Abdelsattar et al., 2023; Chaudhary et al., 2023; Poupin et al., 2023). Compared to other review literature, this study focuses on the impact of soil microorganisms on plants, soil, and plant-soil ecosystems rather than just on the individual study of plants and soil. We consider that microorganisms directly or indirectly affect soil or plants in the process of influencing plants or soil, and the relationship between the two is very close (Jing et al., 2022). This study gives a thorough and organized overview of how microbial communities interact at the phylum and genus levels, and explains the connections between microorganisms, plants, and soil, and stresses how these results can be applied to improving ecosystem functions to fix broken ecosystems and clean up the environment. According to the current study, it was found that a large number of studies about soil microorganisms have investigated the specific effects of specific microbial groups on plants and soil (Trivedi et al., 2021). In this study, we define bacteria, fungi, and archaea as important microbial communities and summarize and clarify the mechanisms by which these three groups influence plants and soil. The research progress on how microorganisms play a role in environmental restoration was illustrated to provide a theoretical reference for future studies in this field. In addition, this study only discusses the effects of important microbial communities such as bacteria, fungi, and archaea on plants and soil separately. The interactions between other microbial communities and the complex relationships between plants and microorganisms that affect plants and soil still need further summarization and deepening, e.g., cyanobacteria and PGPR working together can control nutrient management to boost biomass production and clean up polluted or inappropriate environments (Prasanna et al., 2012). Again, different plant species, like clover, alfalfa, and grasses, affect the diversity and function of rhizobacterial communities, which in turn affects the rate at which PAHs break down to effectively reduce pollution problems in the environment (Wang et al., 2021c).

### 4.2 Prospect and deficiency

Currently, it has been proven that utilizing the mechanisms of microbial action in different fields to promote ecological functions is a scientific, clean, and efficient approach. Next, it is necessary for us to screen and identify more economically and environmentally friendly beneficial microorganisms, expand their applications, promote ecosystem functions, and maintain ecosystem stability. On the other hand, the improvement of molecular information technology remains the greatest challenge and opportunity in current microbial research, which not only helps in identifying and screening more new microbial populations but also aids in predicting the ecological functions of these new populations. Utilizing beneficial ecological functions is crucial for maintaining ecosystem stability and environmental cleanliness. For instance, through genetic engineering combined with other technologies, important genes with microbial functions can be inserted into plants, enabling them to better withstand environmental pressures and protect plant health (Ding et al., 2021; Reboledo et al., 2021). Last but not least, it is significant to figure out how important microbial communities work to ecological functions, which will help us apply these communities to manage the environment and restore ecosystems.

Finally, we acknowledge that there are limitations to this study. Firstly, the definition of important soil microbial communities in this paper is based on their relatively high proportions in the soil, their importance in influencing plants and soil, and their high relevance to the research theme of promoting ecosystem functions. If other soil microbial communities that meet the above-defined criteria are discovered, it may change the scope of this study and require corresponding adjustments to the conclusions. These microbial communities would also serve as complementary, important soil microbial communities in this study. Additionally, there may be some subjectivity in the final selection of literature during the retrieval process, which could introduce certain biases in the conclusions. However, this does not affect the overall direction of development in this field.

## 5 Conclusions

Soil microorganisms play an important role in environmental remediation. Clarifying the mechanisms by which microorganisms influence plants and soil, as well as their applications in environmental remediation, helps us restore and rebuild ecosystems in a green and clean manner, maintaining ecological balance and stability. We used the SLR method to screen 266 articles in this field and reached the following conclusions: (1) Over the past 30 years, the volume and research focus of publications on this topic have shown different changes over time. Overall, there has been a growing trend in the number of publications over the years, but research on archaea has grown more slowly. Studies on the mechanisms of important microorganisms have touched upon microbial physiological activities, the identification of new microbial species, and functional applications. (2) In terms of specific mechanisms, bacteria and fungi promote plant colonization through decomposing soil organic matter, solubilizing elements such as nitrogen, phosphorus, and potassium, symbiosis with plants, secretion of phytohormones, and influencing soil structure, soil development, and plant growth. They also protect plant health by inhibiting pathogens and inducing plant resistance, enhancing the disturbance resistance of the plant-soil ecosystem. Soil archaea degrade low-molecular-weight organic compounds, participate in the cycling of nitrogen, phosphorus, and carbon, and produce plant hormones and antibiotics, promoting plant growth, protecting plant health, and effectively maintaining ecosystem stability. (3) In practical applications, the principles of important microbial communities can be used to remediate contaminated soil and water, reducing environmental pollution. In addition, selected functional microorganisms can be used to produce biofertilizers, biocontrol agents, and pesticides, promoting plant growth, protecting plant health, enhancing the functionality of the plant-soil ecosystem, maintaining ecosystem stability, and improving disturbance resistance. (4) Identifying new microbial species and predicting their functions remain the greatest opportunities and challenges in the current field of microbial environmental remediation. Future research on soil microorganisms should focus on natural environments, explore more beneficial microbial communities, pay particular attention to the coupling of biodiversity and ecological functions, promote the stable enhancement of ecosystem functions, help rebuild and restore damaged ecosystems, and ultimately achieve the goal of environmental remediation.

## Author contributions

XW: Conceptualization, Methodology, Project administration, Software, Validation, Visualization, Writing – original draft. YC: Data curation, Funding acquisition, Resources, Supervision, Writing – review & editing. SS: Conceptualization, Data curation, Project administration, Resources, Supervision, Writing – original draft.
